# A Novel Method for Humidity-Dependent Through-Plane Impedance Measurement for Proton Conducting Polymer Membranes

**DOI:** 10.3390/membranes9050062

**Published:** 2019-05-07

**Authors:** Patrick Heimerdinger, Andreas Rosin, Michael A. Danzer, Thorsten Gerdes

**Affiliations:** 1 Keylab Glasstechnology, University of Bayreuth, 95447 Bayreuth, Germany; andreas.rosin@uni-bayreuth.de (A.R.); thorsten.gerdes@uni-bayreuth.de (T.G.); 2 Chair of Electrical Energy Systems, University of Bayreuth, 95447 Bayreuth, Germany; danzer@uni-bayreuth.de

**Keywords:** through-plane conductivity, humidity-dependent, anisotropic composite membrane, polymer electrolyte membrane

## Abstract

In this study, we introduce a through-plane electrochemical measurement cell for proton conducting polymer membranes (PEM) with the ability to vary temperature and humidity. Model Nafion and 3M membranes, as well as anisotropic composite membranes, were used to compare through plane and in plane conductivity. Electrochemical impedance spectroscopy (EIS) was applied to evaluate the proton conductivity of bare proton exchange membranes. In the Nyquist plots, all membranes showed a straight line with an angle of 60–70 degrees to the *Z*’-axis. Equivalent circuit modeling and linear extrapolation of the impedance data were compared to extract the membrane resistance. System and cell parameters such as high frequency inductance, contact resistance and pressure, interfacial capacitance were observed and instrumentally minimized. Material-related effects, such as swelling of the membranes and indentation of the platinum mesh electrodes were examined thoroughly to receive a reliable through-plane conductivity. The received data for model Nafion and 3M membranes were in accordance with literature values for in-plane and through-plane conductivity of membrane electrode assemblies. Anisotropic composite membranes underlined the importance of a sophisticated measurement technique that is able to separate the in-plane and through-plane effects in polymer electrolytes.

## 1. Introduction

The technology of low temperature fuel cells that work at temperatures below 100 °C is well researched and established in the industry. Typically, Nafion or similar membranes are used, which are chemically very stable and have a high proton conductivity (10^−2^ S·cm^−1^) [1]. Dispersed platinum ensures a high catalytic efficiency for both reactants; hydrogen and oxygen. Although this technology is successfully implemented on laboratory scale or as small stand-alone solution, it still meets the challenge of market penetration. Because the success of this technology is still limited, the focus is on membranes and their further development concerning optimization of stability, efficiency and lifetime. Especially working temperatures of up to 130 °C are desirable for large scale usage [2,3,4,5,6]. For the evaluation and further development of proton exchange membranes, measurements of proton conductivity are the decisive tool. Accordingly, for many membranes [7,8,9,10,11,12,13], in particular Nafion [1,14,15,16,17,18,19], a very large data set is already available, which was generated with electrochemical impedance spectroscopy. However, the data vary from measurement to measurement, so that comparability on a scientifically founded level is almost impossible. The main reason for this is that the resistance measurement is strongly influenced by the technique used and by the geometry of the cell under investigation. The geometrical direction in which the measurement is performed plays a significant role and can be summarized in two cases. An in-plane measurement is easily set up and can be performed with a two- or four-electrode arrangement. Due to the cell configuration, the large cell constant and additional sense electrodes in the four-electrode arrangement, this technique ensures high measurement accuracy [14,20,21,22,23,24]. The more relevant direction though is the route of the proton flow in a fuel cell, that means through the membrane and not in-plane. Through-plane measurement techniques, with a direct contact of membrane and electrodes, are limited to a two-electrode arrangement due to the thickness of the conductive material and the challenge to locate additional electrodes. By this fact, this arrangement suffers from measurement artifacts associated with the measurement cell and not the investigated probe material. Another issue contributes to the probe geometry. A large electrode/electrolyte interface and a relatively short measurement distance induce a low resistance value and a large impact of the interfacial impedance. Few approaches on this technique have been reported in literature [2,14,15,16,22,25,26,27,28,29,30,31]. Mostly the conductivity is examined out of single cell testing [32,33,34,35,36,37] The few reported techniques for through-plane conductivity can be separated into two groups. Once a humidity dependent cell arrangement is introduced, there is always a membrane-electrode assembly used [22,25,26,29]. Techniques or cell arrangements for bare membranes are only used for measurements in water without any temperature or humidity variation [15,28,38].

In this paper, we introduce a cell arrangement that allows a temperature and humidity dependent impedance measurement on bare polymer electrolyte membranes in through-plane direction. We provide a comparison of the conductivity at controlled humidity and temperature for a measurement in the direction of proton passage and in-plane of the membrane. Special emphasis is placed on the analysis and interpretation of the measured resistance values and their conversion to conductivity values.

## 2. Materials and Experimental Arrangement

### 2.1. Materials

Nafion 117 membrane from DuPont and 3M ionomer membranes with an equivalent weight of 825 g/eq in 100 µm thickness were used in this work. All membranes were pretreated referring to described procedures in literature [1,38,39,40]. The membranes were cleansed in 3% aqueous H_2_O_2_ Solution (Merck KGaA, Darmstadt, Germany) for 1 h to remove any residues, rinsed in deionized water several times, soaked in 1.5 M H_2_SO_4_ (Merck KGaA, Darmstadt, Germany) for 1 h to ensure full protonation, and again rinsed in deionized water several times to remove excess acid. All samples were stored at room conditions and soaked in deionized water 24 h prior to use. The ion exchange capacities were 0.90 mmol/g for Nafion 117 and 1.21 mmol/g for 3M D825 according to their equivalent weight.

**Figure 1:**
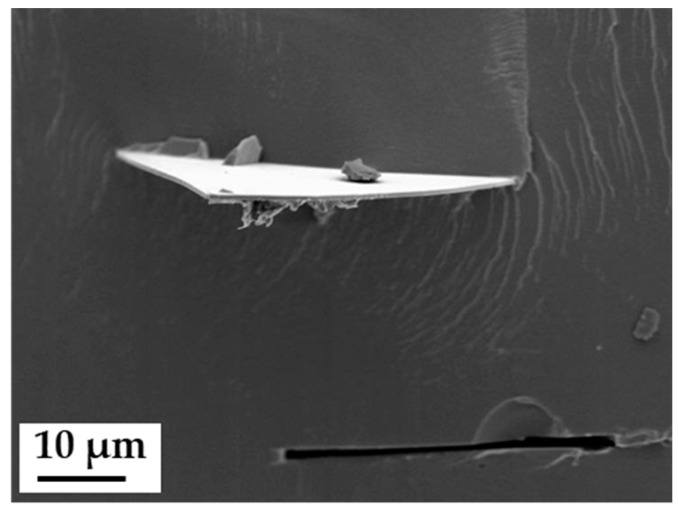
Scanning electron microscope (SEM) micrographs (secondary electrons) of a cryofractured composite membrane with a planar-oriented glass platelet (upper half of picture), and a vacancy of a ripped off platelet (lower half of picture).

Anisotropic composite membranes were synthesized as follows, beginning with the treatment of the filler material. Glass platelets, as anisotropic additives, were synthesized as described by Kyrgyzbaev et al. [41]. To prevent leaching of alkaline ions from the glass into the membrane, the glass platelets were treated in sulfuric acid (96%, Merck KGaA, Darmstadt, Germany) over night and washed several times in deionized water. After the acid treatment, protons replaced the alkaline ions at the surface layer and -OH groups have saturated the glass surface. Ionomer powder (3M 825 EW) was dissolved in diethyleneglycolmonoethylether (Merck KGaA, Darmstadt, Germany), and a screened glass flake fraction (68–125 µm) was added to the slurry to receive an additive content of 5 and 10 weight percent related to ionomer dry mass. Principles of membrane casting, thermal treatment and activation are described in literature. [39,42,43]. The casting was prepared by a doctor blade on a polyimide foil. The doctor blade technique ensured the distinct orientation of the glass platelets in the polymer film [44]. Membrane drying occurred at 80 °C for 24 h (Memmert GmbH, UFE 400, Schwabach, Germany). The dried composite membrane was soaked in 1 M NaOH (Merck KGaA, Darmstadt, Germany) to perform an ion exchange. After several washing steps in deionized water for removal of excess alkalis, the composite membrane was tempered at 180 °C for 30 min and subsequently treated as described above to ensure a full re-protonation. The anisotropic composite membranes were also stored at room conditions and soaked in deionized water 24 h prior to use. A SEM micrograph (Jeol JSM-840A, Freising, Germany; 5 kV accelerating voltage/6 × 10^−11^ A beam current) of a cryofracture of such a composite membrane is shown in Figure 1. The dark area represents the ionomer. A horizontally aligned glass platelet is clearly visible, protruding beyond the fracture face. Right underneath is a vacancy, where another glass platelet was ripped out of the ionomer matrix during cryofracture. The light grey lines on the ionomer are fracture patterns.

### 2.2. Experimental Arrangement and Electrochemical Cells

**Figure 2:**
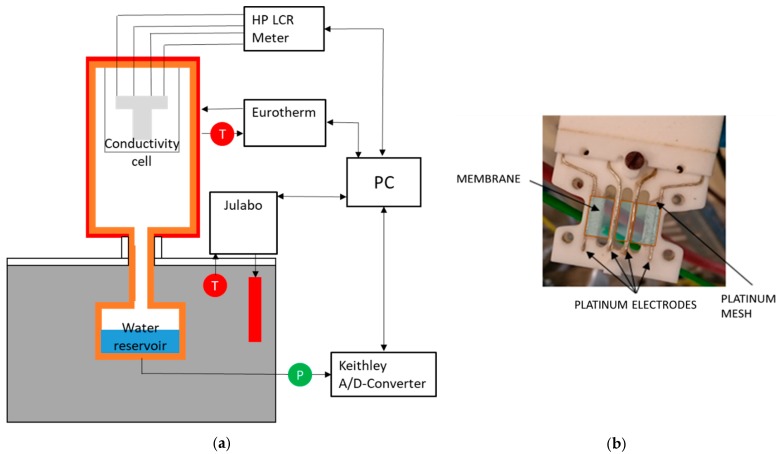
(**a**) Experimental arrangement and (**b**) in-plane measurement cell.

To determine ionic conductivity, potentiostatic impedance measurements were implemented using a Hewlett Packard HP 4284A LCR meter (Keysight Technologies, Santa Rosa, CA, USA) in the range of 20 Hz up to 1 MHz and 20 mV amplitude. Temperature and humidity dependent measurements were carried out in an experimental arrangement in analogy to the one described by Alberti et al. [2]. The arrangement shown in Figure 2a allows temperatures between 70 and 130 °C, in a pressure range from 1 to 4 bar, and humidities between 100 and 20% rh. Each set point for temperature and humidity was kept for 2.5 h to ensure a sufficient equilibration of the membranes. To compare the new designed through-plane conductivity measurement configuration with literature data, additional measurements were performed by placing the cell in deionized water at room conditions.

For in-plane conductivity measurements, the cell shown in Figure 2b was set up according to the four-electrode AC impedance method described in literature [20,21,40,45]. The shown cell is suitable for membranes with 2 cm length and 1 cm width and has a measurement distance of 0.5 cm for the impedance measurements.

**Figure 3:**
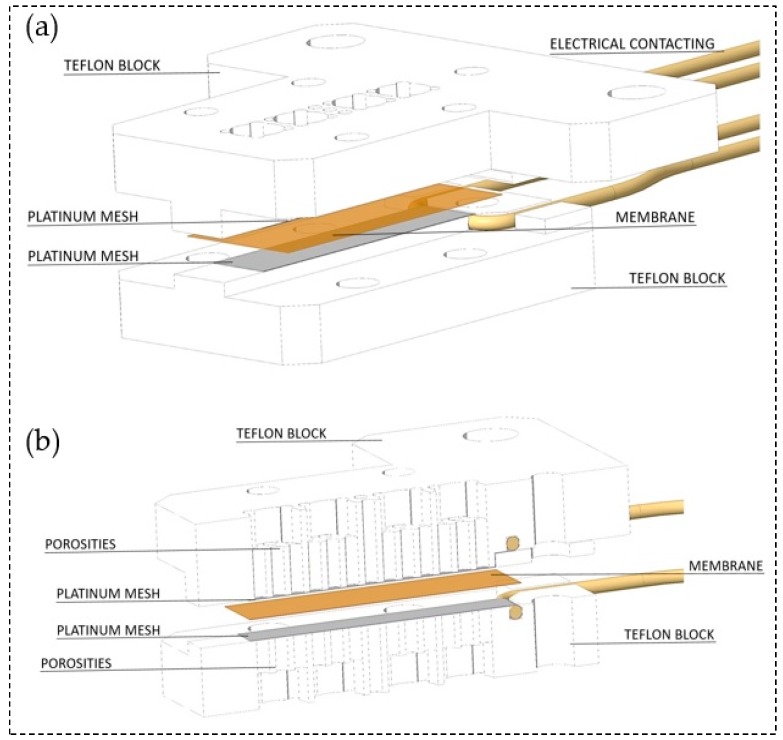
(**a**) Arrangement of the through-plane impedance cell and (**b**) through-plane impedance cell at cross section.

The through-plane conductivity measurement configuration was set up as shown in Figure 3. Herein, two polytetrafluoroethylene (PTFE) blocks build the framework. Both, top and bottom block are perforated in the central segment to ensure air circulation towards the membrane. Two platinum meshes ensure the conduction of the membrane and define the measurement geometry as specified in Table 1. Gold wires attached to the meshes serve as electrical contact inside the experimental arrangement. In order to apply a slight contact pressure four nylon screws fixed the two PTFE blocks. For high contact pressure, additional perforated metal plates and screws were used to support and fix the PTFE scaffold. The cell fits membranes with 2 cm length and 0.5 cm width. It provides a measurement distance for the impedance measurements equal to the thickness of the tested membrane.

In order to improve the accuracy of the impedance measurement, both conductivity cells were connected to the Hewlett Packard HP 4284A LCR meter according to the four-terminal pair (4TP) configuration [46]. The management of the measuring bridge, the temperature controller and thermostats as well as the logging of the measurement data occurred with a software programmed in house. Before each cell was equipped with a membrane, the cell had to be calibrated by means of short compensation to correct high frequency inductance. An accuracy with a test resistance of 0.25 Ω had to provide an accuracy of less than ±1 × 10^−3^ Ω over the whole frequency spectrum to ensure a successful calibration.

For both cell types (in-plane and through-plane), the conductivity was calculated according to following equation:

*σ* = 1/*ρ* = *d*/(*R*·*A*) (1)

where *d* is the membrane thickness, *R* the membrane resistance, and *A* the cross-sectional current carrying area. In case of in-plane measurement, *d* is equal to the distance of the inner electrode pair. In case of through-plane measurement, *d* is equal to the thickness of the membrane. In case of in-plane measurement, *A* is the area cross-section of the ionomer membrane. In case of through-plane measurement, *A* is equal to the contact area of platinum mesh and ionomer membrane.

## 3. Results and Discussion

### 3.1. Interpretation and Evaluation of the Impedance Spectra

**Figure 4:**
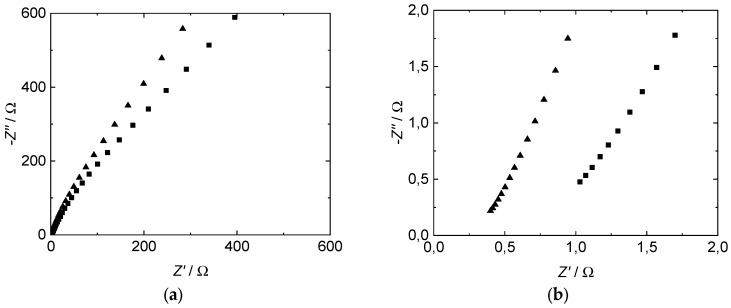
Measured through-plane impedance of Nafion™ 117 (■) and 3M D825 (▲) ionomer membranes, plotted as Nyquist diagram. (**a**) Whole frequency spectrum. (**b**) Zoom.

The impedance spectra of all proton-conducting membranes investigated in our study show a straight line with a slope of ~60–70 degrees in the measured range from 1 MHz to 20 Hz (Figure 4). Compared to the results described in literature, especially by Soboleva et al. [15], we found a slightly lower angle which indicates a difference in the electrode–electrolyte interface between a platinum plate and the used mesh in our case. The straight slope is in good agreement with the concept of a double layer capacitance formation at the interface between electrolytes and metal electrodes at blocking conditions without charge transfer processes [47,48].

**Figure 5:**
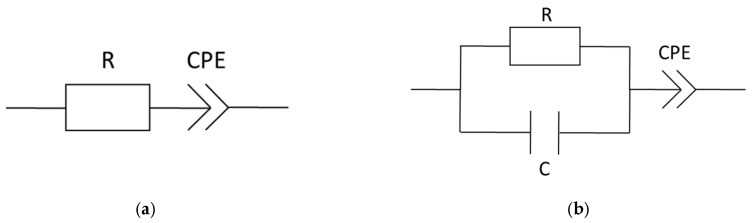
Equivalent circuit models, (**a**) equivalent circuit for comparison to linear extrapolation and (**b**) simple equivalent circuit summarized out of the cited literature above; R = resistance, C = capacity, and CPE = constant phase element.

To determine the membrane resistance from Nyquist plots, there are mainly three common ways described in literature. Linear extrapolation of the measured data down to the *Z*’-axis of the Nyquist plot and accepting the *Z*’-value of the cross section as the membrane resistance is the fastest appraisal [2,8]. In case of impedance measurements up to low two-digit MHz numbers, which express the beginning of a semicircle, those *Z*’ corresponding to the minimum of *Z*’’ can be assigned to the membrane resistance [14,25,49,50]. The more sophisticated method is an equivalent circuit fitting. Several equivalent circuit models are described in literature [15,16,23,26,27,29,30]. The models vary in complexity and composition but all can be assigned to attributes of the membrane and the membrane-electrode interface. A discussion or explanation shall not be part of this study. We selected the following two models (Figure 5) to provide a comparison of extrapolated and modeled resistance values and to give a thought-provoking impulse regarding the variety of conductivity values found in literature. Data processing and equivalent circuit fitting were implemented with a software tool provided in the Supplementary Materials.

Table 2 shows an excerpt of the resistance values determined at varying frequency ranges from a through-plane impedance spectrum. The measurement was carried out at room temperature in deionized water with commercial 3M 825EW and Nafion 117 membranes. Correlation factors for the modeled data are presented in the supplementary. Regarding frequency ranges above 10 kHz, resistance values of tested membranes determined by linear extrapolation and its modeled equivalent circuit (R-CPE) are slightly lower, compared to the values determined by the equivalent circuit model RC-CPE. The discrepancy of the absolute resistance values increases with the thickness of the tested membranes (100 µm for the 3M membrane and 177.8 µm for Nafion), though the ratio of the values stays the same at 1.15:1 equivalent circuit fitting with RC-CPE to linear extrapolation. The R-CPE model provides the lowest values. An increase of the frequency range shows a higher increase of values for the linear extrapolation and R-CPE model, compared to the RC-CPE model. At the widest regarded frequency range (1–10 kHz), the RC-CPE model provides the lowest resistance values. In fact, the absolute resistance values of all three methods strongly depend on the frequency range used for the evaluation, leading to either under- or overestimation of the membranes’ resistance.

**Figure 6:**
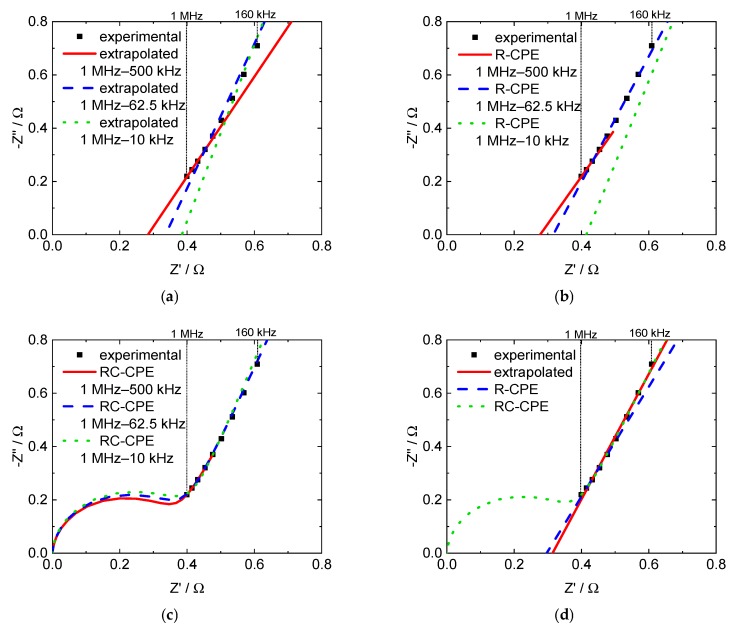
Nyquist plots with extrapolated and modeled through-plane impedance data for a 3M 825 EW membrane, (**a**) linear extrapolation, (**b**) R-CPE model, (**c**) RC-CPE model and (**d**) comparison of all three evaluation methods in the frequency range from 1 MHz to 160 kHz.

Figure 6 exemplarily illustrates the differences in frequency ranges. Figure 6a shows the results achieved with linear extrapolation, Figure 6b with the R-CPE equivalent circuit, and Figure 6c with the RC-CPE equivalent circuit. Figure 6d illustrates a comparison of the three methods in the same frequency range.

In order to prevent an under- or overestimation of the membrane resistance, ohmic resistance and proton conductivity were hereinafter calculated using the linear extrapolation method after performing data processing for a sweep of frequency bands and selecting the best result according to the highest coefficient of determination.

### 3.2. Contact Resistance in Through-Plane Measurement

**Figure 7:**
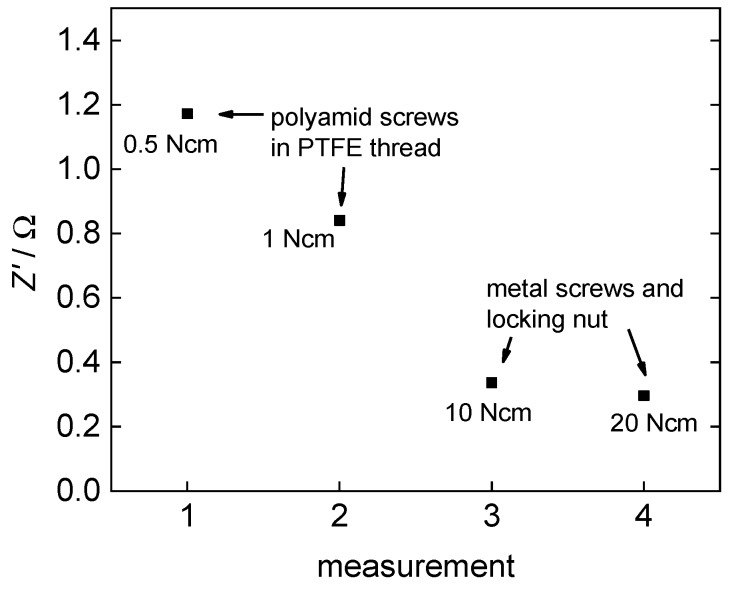
Effect of clamping pressure on the examined resistance of 3M 825 EW membrane.

In light of the fact that in this work, plane polymer electrolyte membranes and no merged membrane electrode assemblies were examined, the quality of the contact area between the membrane and the platinum electrodes is a crucial factor for the impedance measurements. Soboleva et al. [15] described issues and an influence of the clamping pressure on measurements with platinum plates. The first issue they described is due to a water film at the interface between the platinum plates and the membrane. This issue can be neglected here, because a platinum mesh is used instead. The second issue described is due to the morphology of the membranes and was possibly increased by the use of the platinum mesh. The morphology in the contact area may be different to the bulk what leads to a diverse dispersal of the voltage field in the membrane. Examining the effect of the clamping pressure, we observed the same effect. An increasing clamping pressure leads to a decrease in *Z*’ as shown in Figure 7.

At first, *Z*’ drops very fast with an increasing clamping pressure but then converges to an almost constant domain where additional pressure increase does not show any further effect. Overall, *Z*’ drops from 1.2 Ω at 0.5 N·cm torque to 0.3 Ω at 20 N·cm torque.

### 3.3. Effects of the Platinum Mesh on Humidity-Dependent through-Plane Conductivity

**Figure 8:**
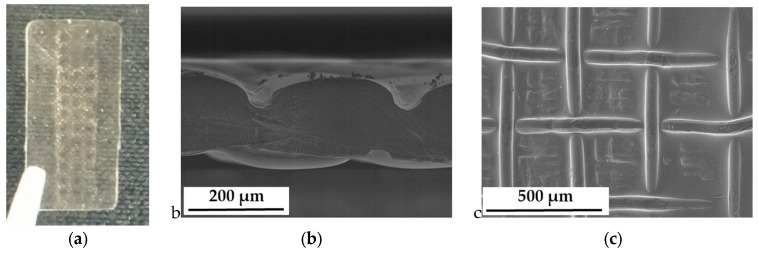
Indentations of the Pt mesh into the electrolyte membrane; (**a**) Nafion 117 after disassembly; (**b**) SEM micrograph of a cross section; and (**c**) SEM micrograph of the electrolyte surface.

The use of an open platinum mesh instead of a plate ensures a consistent air supply towards the membrane surface at both electrodes and a subsequent equilibration of the electrolyte membrane to defined humidities. In addition to the above-described influence of the analyzed frequency range and clamping pressure, we realized the necessity to examine the thickness and topology of the membranes in the compressed state. Generally, we observed two different cases:

1. After conductivity measurements in water at room temperature, we did not observe any changes in membrane thickness or topology.
2. After conductivity measurements at elevated temperature and in humidified air (<100% rh) we observed clearly visible mesh indentation after cell disassembly (Figure 8).

We assume that complete swelling of the membrane in water increased elasticity, which made the membranes resistant to any indentation of the platinum mesh, even with increasing clamping pressure. The increase in membrane conductivity can be associated with a better membrane/electrode interphase and a decreased contact resistance. On the other hand, humidity dependent conductivity measurements left mesh indentations in the membranes. We assume that the reduction of absolute water uptake and the elevated temperature softens the membrane, i.e., increasing its plasticity. Under the clamping pressure, the platinum mesh immerses into the polymer, leaving such indents as shown in Figure 8.

**Figure 9:**
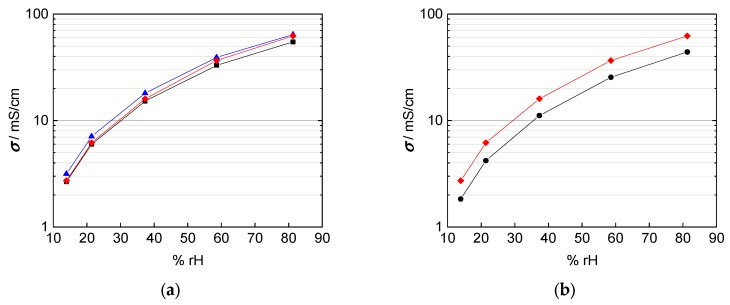
Calculated conductivity of Nafion 117 at 100 °C with (**a**) black: thickness of dry membrane; blue: swollen thickness; red: interpolated thickness with respect to humidity; and (**b**) black: interpolated thickness with respect to humidity and mesh indentation; red: interpolated thickness with respect to humidity.

According to the fact that the calculation of the conductivity strongly relies on the electrode distance for through-plane measurements, the deformation of the membrane under the influence of humidity and temperature is crucial for this type of measurement. Sorption isotherms for Nafion 117 and 3M 825 EW membranes are explicitly described in literature [51,52,53,54,55]. Regarding the humidity range of the conductivity measurements, the water uptake of the electrolyte membranes is linear with humidity. Therefore, the thickness of the membranes was calculated subsequently with a linear fit between the swollen and dry state. Measured and calculated values for Nafion 117 are displayed in Table 3. The thickness was measured as distance between the mesh indentations as shown in Figure 8b. Resulting humidity dependent proton conductivity is shown in Figure 9.

**Figure 10:**
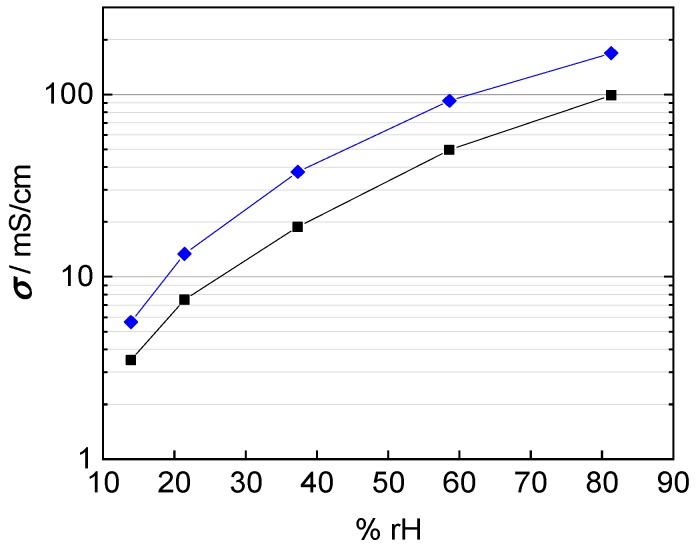
Calculated conductivity of Nafion 117 at 100 °C; blue: correction for humidity dependent membrane thickness and open mesh area; and black: correction for humidity dependent membrane thickness and immersed mesh area.

In fact, the calculated conductivity values are lower than those reported in literature, which vary from 80 mS/cm up to 140 mS/cm at 100 °C and 100% rh [1,2,20,22,25]. The conductivities shown in Figure 9 were calculated with a conducted area between membrane and electrodes corresponding to the enclosed area of edge length, i.e., *A* = *a* × *b*. The mismatch in conductivity and the obvious mesh indentations let us reconsider the influence of the contact area of the platinum mesh, as the open mesh surface is 65%. Regarding Equation (1) for conductivity calculation, the open mesh surface had to be taken into account. The resulting conductivity values with respect to the open mesh surface and the mesh indentation thickness are illustrated with help of the blue line in Figure 10. Conductivity values increased due to the adapted contact area, with *A*_1_ = 0.35·A, in the denominator of Equation (2).

(2) $$\sigma =\frac{1}{\rho }=\frac{d}{R\cdot A_{1}}$$

**Figure 11:**
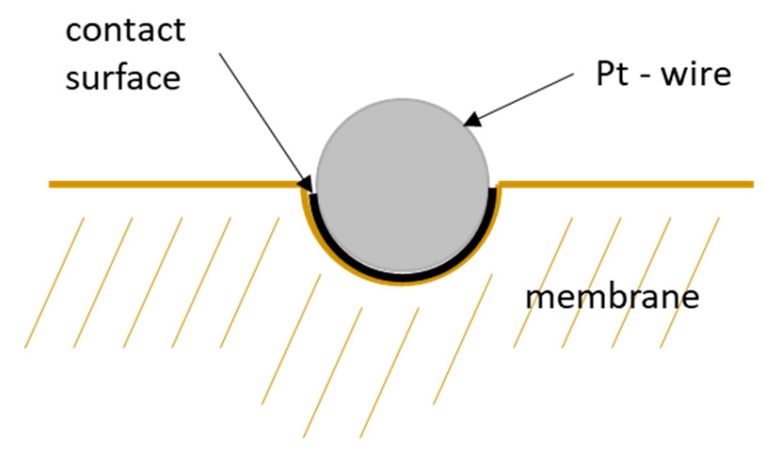
Schematic illustration of the contact surface of a platinum wire of the mesh.

However, this correction considers wire diameter and spacing only. That means the calculation above is based on the projected surface area of the mesh. Hence, it does not consider the immersion of the platinum mesh into the membrane and underestimates the actual contact area. Figure 11 illustrates the area of contact if the platinum mesh immerses into the membrane. The current-carrying surface should be calculated according to mesh specifications listed in Table 1.

(3) $$A_{s}=\left(1–A_{2}\right)$$

(4) $$A_{2}={\left(\frac{w}{p}\right)}^{2}{\;}^{\;}$$

where $A_{s}$ expresses the overall contact surface, $A_{2}$ stands for the open sieve surface, w is the mesh opening and p the nominal size of the wire separation estimated with half of the wire circumference (U/2=π·d/2) instead of the wire diameter. The corrected proton conductivity is shown as black line in Figure 10. It is in good accordance with conductivity described in literature [1,26]. Finally, the interphase area was determined to be

(5) $$A_{interphase}=0.472\;\times \;A$$

### 3.4. In-Plane vs. Through-Plane Conductivity in Water and Humidity-Dependent

Various groups in literature report anisotropy of in-plane and through-plane conductivity in polymer electrolyte membranes. For example, Gardner et al. [14,30] described for Nafion 117 a disparity of as much as 70%. The authors reported higher values for in-plane conductivity. They explained the anisotropic effect with the orientation of conducting channels along the direction of membrane extrusion and the alignment of ionic clusters at the membrane/electrode interface. Soboleva et al. [15] confirmed the anisotropy for several Nafion membranes though they found a maximum anisotropy of 40% for Nafion 112 and 20% for Nafion 117 but no anisotropy for a Nafion 211 membrane. In contrast, Yamada et al. [56] observed anisotropic conductivity with higher values for through-plane conductivity.

**Figure 12:**
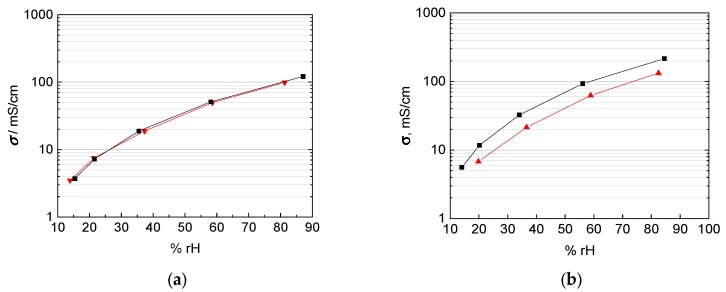

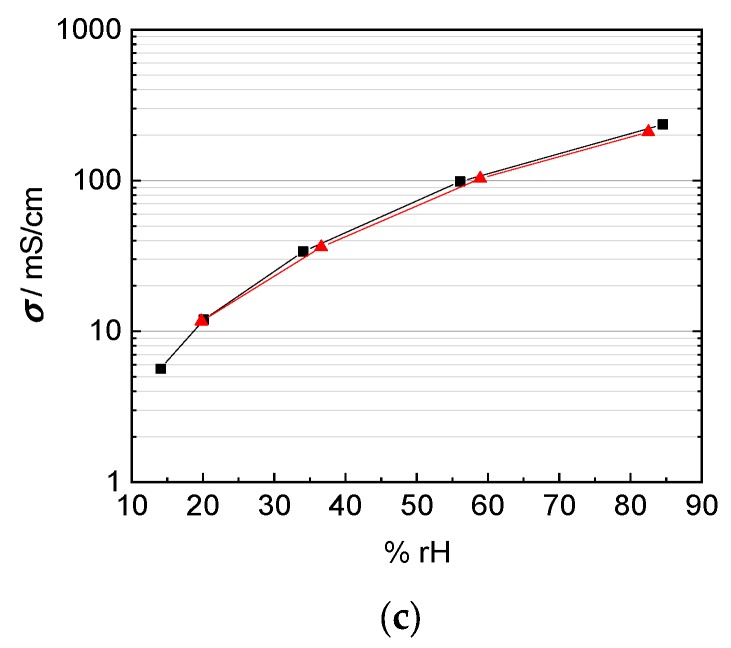
Comparison of in-plane (black) and through-plane (red) proton conductivity of (**a**) Nafion 117, (**b**) 3M 825 EW membrane with 100 µm thickness, and (**c**) 3M 825 EW membranes with 200 µm thickness.

Taking into account the corrected interphase area as described above, our results indicate that the correction of interphase area and membrane thickness is more relevant than any anisotropy effect for the casted membranes investigated here. The Nafion 117 membrane in Figure 12a shows almost the same conductivity over the humidity range, independent of in-plane or through-plane direction. The in-plane conductivity was calculated with help of the humidity dependent thickness of the membrane as described above. The through-plane conductivity was calculated, as described above, in dependence of the humidity dependent thickness and of mesh indentation with corrected interphase area. The thinner 3M 825 EW membrane with 100 µm thickness, shown in Figure 12b shows a maximum derivation of 40% between in-plane and through-plane direction.

This drift in the accuracy of the through plane measurement technique with thinner membranes can be explained with the increasing influence of the cell constant once the membrane thickness decreases [14,20,21,22,23]. Anisotropic effects with the orientation of the conducting channels and the alignment of ionic clusters at the membrane/electrode interface as described above may apply as explanation for the extruded Nafion 117 membrane, but not for the casted 3M membranes.

Matos et al [25] and Slade et al [1] describe a linear increase of the membrane resistance and the number of membranes or the membrane thickness respectively. The through-plane conductivity by mounting two 100 µm 3M 825 EW membranes with an overall thickness of 200 µm is shown in Figure 12c. In-plane and through-plane conductivity are equal for the casted 3M membranes where no anisotropy is expected and described in literature.

**Figure 13:**
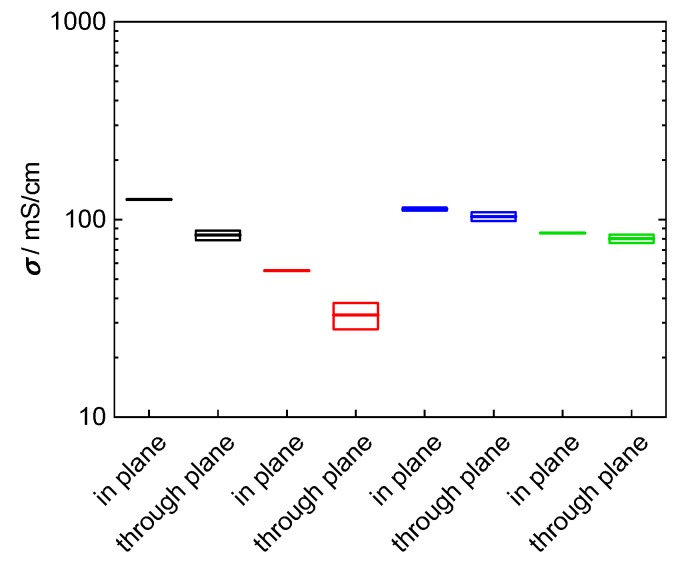
Comparison of in-plane and through-plane conductivity at room temperature in water: black: 3M 825 EW with 5 wt% glass platelets; red: 3M 825 EW with 10 wt% glass platelets; blue: 3M 825 EW (100 µm); and green: Nafion 117.

The objective of this study was to evaluate a new measurement arrangement, to describe the analytical proceeding to extract values for ionic conductivity from impedance data and to demonstrate its necessity. To clarify this aim, two composite membranes were prepared based on the 3M 825 EW ionomer with anisotropic fillers. The used glass platelets show a desired aspect ratio with a large plane surface, 5000–15,000 µm², and a small thickness, 1 to 2 µm. According to the casting process of the membrane, the glass platelets are aligned in direction of the membrane, i.e., they should cause a blocking effect for proton migration in through-plane direction. Our investigations were carried out with membranes filled with 5 wt% (4.25 vol%) glass platelets, and 10 wt% (8.5 vol%) glass platelets, respectively. The results for in-plane and through-plane conductivity for bare and composite membranes are shown in Figure 13. The through-plane conductivity decreased indeed compared to the bare 3M membrane. Through-plane conductivity drops from 100 mS/cm to 80 mS/cm at 5 wt%, to 35 mS/cm at 10 wt%. Hence, the glass platelets block proton transfer efficiently even though their volumetric proportion is less than ten percent. Surprisingly, in-plane conductivity increased from 100 mS/cm for the bare membrane to 120 mS/cm for the composite membrane with 5 wt% fillers, while it drops to 55 mS/cm at 10 wt% filling. Probably in-plane conductivity increased at lower glass platelet filling due to surface conductance in electrolytic environment [57,58].

## 4. Conclusions

Conductivity measurements from various research groups on ionomer membranes like Nafion for PEM fuel cells have already shown that anisotropic conductivity might occur, most likely due to the process of membrane fabrication. Our interest focused on membranes with deliberately introduced anisotropic behavior. Therefore, we proposed a new cell design, which allows temperature and humidity dependent impedance measurements on polymer electrolyte membranes in through-plane direction. This set-up allows determining the proton conductivity in direction of proton migration in operating fuel cells. The reference measurement cell always uses in-plane direction. That means the conductivity measurement might overlook the effect of membrane fabrication or anisotropy. Hence, and interpretation of conductivity data might lead to misinterpretation regarding fuel cell design.

In order to achieve low contact resistance between electrode and membrane the ionomer membrane is clamped and pressed to 20 N·cm. Platinum meshes are used as electrodes, which allow free convection of air and water vapor towards the membrane. We recognized the necessity to take into account the change of membrane thickness in dependence of water uptake, and the immersion of the platinum mesh into the ionomer during the measurement by ex-situ calibration of the cell constant. The ohmic resistance of the membranes was calculated from frequency-dependent impedance measurements using linear extrapolation and equivalent circuit fitting, respectively. Even though latter method allows a more flexible adoption to the impedance curve progression, we found that linear extrapolation was accurate enough for determination of the ohmic resistance of ionomer membranes. However, the selected frequency range is crucial for both methods. A wide frequency band leads to underestimated ohmic resistance; a too narrow frequency band leads to overvaluation. The best practice evaluates the results from different frequency sweeps to identify the best frequency range for data processing.

Taking into account the best practice for both, geometric calibration and impedance measurement, we could finally determine the through-plane conductivity of two different ionomers and validate the measured data with literature data. However, geometric calibration is a crucial point regarding reproducibility and accuracy for this new cell type. In fact, the accuracy of measurement is better for a membrane thickness of 200 µm rather than 100 µm due to the increasing influence of the cell factor. Finally, we could confirm that in contrast to extruded membranes, casted ionomer membranes have almost the same in-plane conductivity as through-plane conductivity.

Secondly, we investigated the conductivity of ionomer membranes filled with glass platelets in order to raise the level of anisotropy. As expected the ratio of in-plane to through-plane conductivity decreased significantly (50% to 70%). In addition, we observed a decrease of the overall conductivity with increasing filling level, while the anisotropic effect was preserved.

The proposed cell design provides a new and reliable way for determining the anisotropic effects of membrane conductivity, especially when using doped or grafted ionomer membranes for fuel cell applications.

## Figures and Tables

**Table 1:** Cell characteristics for through-plane measurements.

Pt Mesh		PerForation
Characteristics	Conducted Area	
99.9% Pt 0.06 mm wire diameter 0.25 mm nom. Opening 65% open surface 82 × 82 wires/inch	5 × (15–20 mm)	39 × 1 mm diameter air holes

**Table 2:** Comparison of extrapolated and fitted resistance values for Nafion 117 and 3M membranes.

Frequency Range	*Z*‘ 3M 825 EW (100 µm)/Ω			*Z*‘ Nafion 117/Ω		
	Linear Extrapolation	R-CPE	RC-CPE	Linear Extrapolation	R-CPE	RC-CPE
1 MHz–500 kHz	0.284	0.277	0.331	0.729	0.699	0.813
1 MHz–160 kHz	0.314	0.297	0.339	0.793	0.764	0.832
1 MHz–100 kHz	0.325	0.306	0.344	0.817	0.782	0.846
1 MHz–62.5 kHz	0.336	0.316	0.349	0.841	0.806	0.860
1 MHz–10 kHz	0.385	0.414	0.366	0.943	0.848	0.905

**Table 3:** Measured and interpolated thickness for Nafion 117 at different moisture conditions with and without mesh indentation.

Membrane Condition	Thickness with Mesh Indentation	Thickness without Mesh Indentation
dry	120 µm ± 2 µm (measured)	177.8 µm (manufacturer information)
swollen	211 µm ± 3 µm (measured)	211 µm ± 3 µm (measured)
81.3% rH	145 µm (interpolated)	205 µm (interpolated)
58.6% rH	137.4 µm (interpolated)	197 µm (interpolated)
37.3% rH	130.2 µm (interpolated)	187 µm (interpolated)
21.4% rH	124.9 µm (interpolated)	184 µm (interpolated)
13.9% rH	122.4 µm (interpolated)	182 µm (interpolated)

## Supplementary Materials

The following are available online at https://www.mdpi.com/2077-0375/9/5/62/s1, Table S1: Extension of Table 2 with coefficient of determination (*R*^2^) and Sum of Squared Errors (SSE) values. “ec-idea” is a tool for electro chemical impedance analysis of frequency-domain impedance data. It is provided by the Chair of Electrical Energy Systems of the University of Bayreuth under following link: https://www.ees.uni-bayreuth.de/en/ec-idea/index.html.
