# Supplementary material for: A Novel Method for Humidity-Dependent Through-Plane Impedance Measurement for Proton Conducting Polymer Membranes
Source: Membranes (Basel). 2019 May 7;9(5):62. doi: 10.3390/membranes9050062 (PMC6571940; doi:10.3390/membranes9050062)
Supplement: Supplementary file 1 [file membranes-09-00062-s001.pdf]

Supplementary Materials: A Novel Method for Humidity-Dependent Through-Plane Impedance Measurement for Proton Conducting Polymer Membranes

Table S1. Extension of Table 2 with coefficient of determination (R^2) and Sum of Squared Errors (SSE) values.

Frequency range	*Z′* 3M 825 EW (100 μm) / Ω							
	**Linear extrapolation**	**R^2**	**R-CPE**	**SSE**	**R^2**	**RC-CPE**	**SSE**	**R^2**
1 MHz–500 kHz	0.284	0.9974	0.277	1.6453e^{-3}	5.4443e^{-1}	0.331	2.4973e^{-6}	8.9936e^{-1}
1 MHz–160 kHz	0.314	0.9954	0.297	8.8141e^{-3}	6.7094e^{-1}	0.339	8.6546e^{-5}	9.4095e^{-1}
1 MHz–100 kHz	0.325	0.995	0.306	1.3357e^{-2}	7.4654e^{-1}	0.344	2.8739e^{-4}	9.5688e^{-1}
1 MHz–62.5 kHz	0.336	0.9948	0.316	1.9412e^{-2}	8.0634e^{-1}	0.349	7.1283e^{-4}	9.6951e^{-1}
1 MHz–10 kHz	0.385	0.9947	0.414	3.8532e^{-2}	9.7395e^{-1}	0.366	5.2647e^{-3}	9.6212e^{-1}
**Frequency range**	***Z′* Nafion 117 / Ω**							
	**Linear extrapolation**	**R^2**	**R-CPE**	**SSE**	**R^2**	**RC-CPE**	**SSE**	**R^2**
1 MHz–500 kHz	0.729	0.9987	0.699	5.5880e^{-4}	5.1799e^{-1}	0.813	6.7648e^{-6}	8.7581e^{-1}
1 MHz–160 kHz	0.793	0.9969	0.764	2.9908e^{-3}	8.0555e^{-1}	0.832	8.8658e^{-5}	9.2738e^{-1}
1 MHz–100 kHz	0.817	0.9966	0.782	4.6841e^{-3}	8.5855e^{-1}	0.846	2.4149e^{-4}	9.4970e^{-1}
1 MHz–62.5 kHz	0.841	0.9965	0.806	6.6807e^{-3}	9.2486e^{-1}	0.860	5.6803e^{-4}	9.6075e^{-1}
1 MHz–10 kHz	0.943	0.997	0.848	1.9761e^{-2}	9.6376e^{-1}	0.905	4.3186e^{-3}	9.3072e^{-1}
